# Different Contributions of Dyslipidemia and Obesity to the Natural History of Type 2 Diabetes: 3-Year Cohort Study in China

**DOI:** 10.1155/2019/4328975

**Published:** 2019-03-04

**Authors:** Lu Liu, Xiaoling Guan, Zhongshang Yuan, Meng Zhao, Qiu Li, Xu Zhang, Haiqing Zhang, Dongmei Zheng, Jin Xu, Ling Gao, Qingbo Guan, Jiajun Zhao, the REACTION Study Group

**Affiliations:** ^1^Department of Endocrinology, Shandong Provincial Hospital Affiliated to Shandong University, 250021, China; ^2^Shandong Clinical Medical Center of Endocrinology and Metabolism, Institute of Endocrinology and Metabolism, Shandong Academy of Clinical Medicine, 250021, China; ^3^Department of Senior Officials Health Care, China-Japan Friendship Hospital, 100029, China; ^4^Department of Endocrinology, Shandong Provincial Qianfoshan Hospital, Shandong University, 250014, China; ^5^Department of Epidemiology and Biostatistics, School of Public Health, Shandong University, 250012, China; ^6^Scientific Center, Shandong Provincial Hospital Affiliated to Shandong University, 250021, China; ^7^Shanghai Jiao Tong University School of Medicine, China

## Abstract

**Aim:**

It is known that different stages of type 2 diabetes represent distinct pathophysiological changes, but how the spectrum of risk factors varies at different stages is not yet clarified. Hence, the aim of this study was to compare the effect of different metabolic variables on the natural history of type 2 diabetes.

**Methods:**

A total of 5,213 nondiabetic (normal glucose tolerance (NGT) and prediabetes) Chinese older than 40 years participated this prospective cohort study, and 4,577 completed the 3-year follow-up. Glycemic status was determined by standard oral glucose tolerance test both at enrollment and follow-up visit. Predictors for conversion in glycemic status were studied in a corresponding subcohort using the multiple logistic regression analysis.

**Results:**

The incidence of prediabetes and diabetes of the cohort was 93.6 and 42.2 per 1,000 person-years, respectively. After a 3-year follow-up, 33.1% of prediabetes patients regressed to NGT. The predictive weight of body mass index (BMI), serum triglyceride, total cholesterol, and systolic blood pressure in different paths of conversions among diabetes, prediabetes, and NGT differed. Specifically, BMI was the strongest predictor for regression from prediabetes to NGT, while triglyceride was most prominent for onset of diabetes. One SD increase in serum triglyceride was associated with a 1.29- (95% CI 1.10–1.52; *P* = 0.002) or 1.12- (95% CI 1.01–1.27; *P* = 0.039) fold higher risk of diabetes for individuals with NGT or prediabetes, respectively.

**Conclusion:**

Risk factors for different stages of diabetes differed, suggesting personalized preventive strategies for individuals with different basal glycemic statuses.

## 1. Introduction

In the face of the burden of diabetes worldwide [1, 2], great efforts have been made to explore the therapeutics for diabetes, and there have been improvements in the prognosis of diabetes [3]. However, this advance is overshadowed by the unprecedented growth in the numbers of people with diabetes [4, 5]. Thus, preventing the onset of diabetes is indeed the cost-effective strategy in disposing of the burden of diabetes [6, 7].

Generally, over the disease course of type 2 diabetes, individuals progress from normal glucose tolerance (NGT) to prediabetes (isolated impaired fasting glucose (i-IFG), isolated impaired glucose tolerance (i-IGT), or combined status of IFG and IGT (IFG-IGT)) and finally develop to overt diabetes [8]. On the other hand, a substantial number of people with prediabetes spontaneously regress to NGT over time, and this regression is associated with a significantly reduced risk of future diabetes and its complications [9]. Therefore, a full evaluation on how the spectrum of risk factors varies at the different stages of pathophysiology of type 2 diabetes is indispensable for guidance of targeted preventive strategies.

Longitudinal investigations of predictors for glycemic outcomes have been conducted in various populations of nondiabetic subjects. However, previous studies focused on either the identification of risk factors for the deterioration from NGT or prediabetes to overt diabetes [10–13] or the association with the reversion from prediabetes to NGT [14, 15]. And there is a lack of study investigating the risk factors for the two opposite outcomes in the same population. Indeed, such an approach can help to understand how the spectrum of risk factors varies at the dynamic stages of type 2 diabetes and thus provide data that is more informative for directing more precise preventive strategies for diabetes.

The Risk Evaluation of cAncers in Chinese diabeTic Individuals: a lONgitudinal study (REACTION) was a multicenter, prospective, observational cohort study conducted to evaluate chronic diseases in middle-aged to elderly Chinese people [16]. Using data collected as part of the REACTION study, the present study aimed to (1) observe the change in glycemic status of nondiabetic subjects and estimate the incidence rates of diabetes and prediabetes and (2) evaluate the risk factors for progression from NGT or prediabetes to diabetes and the predictors for interconversion between prediabetes and NGT, over a 3-year follow-up.

## 2. Subjects, Materials, and Methods

### 2.1. Study Population

The REACTION study was a noninterventional cohort study which enrolled 259,657 Chinese people (≥40 years of age) from 25 communities in mainland China between 2011 and 2012 with follow-ups planned at 3, 5, and 10 years [16]. Data of one of the 25 communities, in Ningyang County, Shandong Province, were selected for this study. Overall, 7,068 subjects participated in the baseline survey. Individuals with diabetes (*n* = 1,016), either self-reported previous diagnosis or detected by the standardized oral glucose tolerance test (OGTT) performed at the baseline survey, were excluded from this study. We also excluded individuals with missing vital data, such as age, gender, or results of OGTT (*n* = 205); individuals with malignant tumors (*n* = 29) or serious liver (either alanine aminotransferase or aspartate aminotransferase higher than 100 U/L) or renal dysfunction (creatinine higher than 105 *μ*mol/L and a glomerular filtration rate below 60 mL/min) (*n* = 78); and individuals receiving medications in the three months prior to the baseline survey that affect lipid metabolism or blood pressure (*n* = 527), including statins, fibrates, angiotensin-converting enzyme inhibitors, angiotensin receptor blockers, *β*-adrenoceptor blockers, calcium channel blockers, or diuretics. Ultimately, 5,213 individuals with NGT or prediabetes were eligible for the current study.

The study protocol conformed to the 1975 Declaration of Helsinki and was approved by the Committee on Human Research at Ruijin Hospital, Shanghai Jiao Tong University School of Medicine. All study participants provided written informed consent.

### 2.2. Follow-Up and Study Measurements

The 3-year follow-up visit was conducted in 2014–2015. Of the 5,213 individuals eligible for this study, 64 died before the 3-year follow-up survey. Among those individuals who were alive, 572 individuals were lost to follow-up, leaving a total of 4,577 individuals (2,833 individuals with NGT and 1,744 with prediabetes at Baseline) who completed the 3-year follow-up survey and were finally involved in the current analysis. The selection of study participants is illustrated in Supplementary Material, Supplementary Figure 1.

Study measurements included detailed questionnaires; clinical and biochemical measurements were collected both at Baseline and at the 3-year follow-up visit. Details are provided in the Supplementary Material, Study Measurements.

### 2.3. Glycemic Status Assessment

Participants' glycemic status at Baseline and 3-year follow-up was determined by standardized OGTT and was classified according to the World Health Organization (WHO) 1999 criteria [17]: NGT, FPG < 6.1 mmol/L and 2hPG < 7.8 mmol/L; i-IFG, FPG between 6.1 and 7.0 mmol/L and 2hPG < 7.8 mmol/L; i-IGT, FPG < 6.1 mmol/L and 2hPG between 7.8 and 11.1 mmol/L; IFG-IGT, FPG between 6.1 and 7.0 mmol/L and 2hPG between 7.8 and 11.1 mmol/L; and diabetes, FPG ≥ 7.0 mmol/L and/or 2hPG ≥ 11.1 mmol/L. i-IFG, i-IGT, and IFG-IGT are three categories of prediabetes.

### 2.4. Statistical Analysis

Outcome rates were estimated by dividing the number of events by the number of persons at risk. The incidence of diabetes and prediabetes per 1,000 person-years with 95% CI was calculated using the number of persons developing these conditions at 3-year follow-up as the numerator and the total person-years as the denominator. Person-years were calculated from the date of the baseline survey until the diabetes or prediabetes occurred or until the 3-year follow-up survey, whichever came first. Age- and sex-standardized estimates of incidence were calculated using the direct method, taking the 2010 census of the Chinese rural population aged 40–79 years as the standard. Quantitative characteristics of the cohort were expressed as mean ± standard deviation (SD) or median (interquartile range) according to their distributions, which were judged by histogram. Categorical data were presented as a number (percentage). One-way ANOVA or the Mann-Whitney *U* test was used to test differences between continuous variables among groups. Differences in categorical data were evaluated by the chi-squared test.

Multivariate logistic regression analysis was used to evaluate the association between baseline clinical characteristics and conversions in glycemic status. The variables that were clinically relevant or had a *P* value < 0.2 in univariate analysis were included as covariables in the multivariate model. Finally, age, sex, family history of diabetes, smoking status, drinking status, physical inactivity, FPG (for individuals with NGT at Baseline) or categories of prediabetes (for individuals with prediabetes at Baseline), BMI, serum total cholesterol (TC), serum triglyceride (TG), and systolic blood pressure (SBP) were entered into the model. BMI, TC, TG, and SBP were categorized according to their values, and odds ratios (ORs) were calculated as the ratio of each category to the reference group. BMI was categorized as <24 kg/m^2^ (normal), 24–28 kg/m^2^ (overweight), or ≥28 kg/m^2^ (obese) [18]; TC was grouped as <5.18 mmol/L (normal), 5.18–6.19 mmol/L (marginally elevated), or ≥6.19 mmol/L (elevated) [19]; TG was grouped as <1.70 mmol/L (normal), 1.70–2.25 mmol/L (marginally elevated), or ≥2.25 mmol/L (elevated) [19]; and SBP was categorized as <140 mmHg (normal) or ≥140 mmHg (high) [20]. ORs per one SD change in baseline BMI, TC, TG, and SBP were calculated to identify the variables with the strongest effect on each path of conversion.

A two-tailed value of *P* < 0.05 was regarded as significant. All statistical analyses were performed using SPSS version 22.0 for Windows (Chicago, IL, USA).

The datasets generated during and/or analysed during the current study are available from the corresponding author on reasonable request.

## 3. Results

Of the 5,213 individuals eligible for this study, 4,577 completed the 3-year follow-up and were involved in this analysis. The baseline characteristics of the individuals who completed the 3-year follow-up and those lost to follow-up were not different (Supplementary Material, Supplementary Table 1). The median follow-up was 3.1 years (range 2.8–3.3 years).

### 3.1. Conversions in Glycemic Status of the Participants

Of the 2,833 individuals with NGT at Baseline, 28.7% progressed to prediabetes and 6.6% developed to diabetes after a median follow-up of 3.1 years (Supplementary Material, Supplementary Table 2). Interestingly, individuals with NGT were more likely to progress to i-IGT (21.5%) than i-IFG (3.4%).

Among the study participants with prediabetes at Baseline, 21.1% progressed to diabetes and 33.1% reverted to NGT (Supplementary Material, Supplementary Table 2). A total of 314 subjects had IFG-IGT at Baseline, of whom 19.4% reverted to NGT and 30.3% developed diabetes at the 3-year follow-up, representing the highest rate of progression to diabetes and the lowest rate of reversion to NGT among the three categories of prediabetes. Individuals with i-IFG at Baseline were more likely to progress to more advanced stages of dysglycemia (IFG-IGT or diabetes) (34.2% vs. 23.2%; *P* < 0.001) and less likely to revert to NGT (32.0% vs. 39.4%; *P* = 0.004), compared with subjects with i-IGT.

### 3.2. Incidence Rates of Prediabetes and Diabetes

Overall, the age- and sex-standardized incidence of diabetes for the entire cohort was 42.2 per 1,000 person-years (Table 1). The incidence rates of prediabetes and diabetes among individuals with NGT were 93.6 and 24.2 per 1,000 person-years, respectively, representing a total dysglycemia conversion rate of 117.8‰. Of all the individuals with prediabetes, the incidence of diabetes was 70.3 per 1,000 person-years. The progression rates from i-IFG, i-IGT, and IFG-IGT to diabetes were 2.9, 2.4, and 4.3 times higher, respectively, than the progression rate from NGT to diabetes.

### 3.3. Baseline Characteristics of the Participants

Participants who progressed from NGT to prediabetes or diabetes had less metabolically favorable clinical and biochemical baseline characteristics compared with those who maintained NGT, including older age, higher BMI, waist, serum TC, TG, low-density lipoprotein cholesterol (LDL-C), blood pressure, FPG, 2hPG, and haemoglobin A_1c_ (HbA_1c_) (*P* < 0.05 for all, Supplementary Material, Supplementary Table 3). Individuals with prediabetes at Baseline who developed diabetes by the 3-year follow-up were older in age and had higher baseline SBP, FPG, 2hPG, and HbA_1c_, compared with those who maintained prediabetes. Unsurprisingly, participants who regressed from prediabetes to NGT had lower BMI, serum TC, TG, LDL-C, FPG, 2hPG, and HbA_1c_ compared with those who maintained prediabetes (*P* < 0.05 for all, Supplementary Material, Supplementary Table 3).

### 3.4. Serum TG Was the Strongest Metabolic Risk Factor for Incident Diabetes

**Figure 1:**
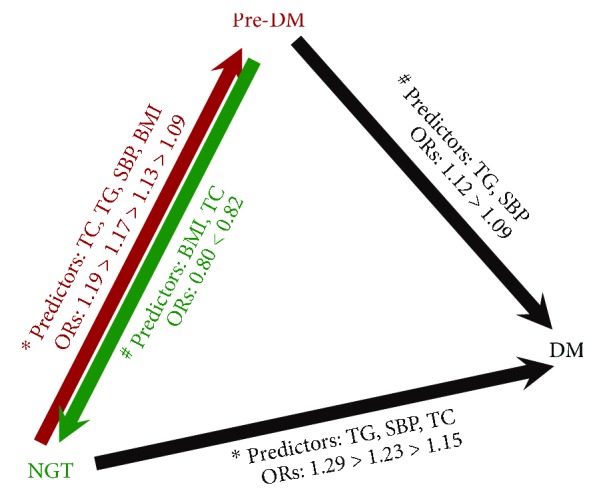
Metabolic risk factors for the conversions between different glycemic statuses. NGT, pre-DM, and DM were the three categories of glycemic status; arrows represent the directions of conversion between different glycemic statuses; factors on each arrow were the independent predictors for each path calculated by multivariate logistic regression analysis. ^∗^Variables in the multivariate logistic regression model: sex, age, family history of diabetes, smoking, drinking, physical inactive, BMI, TC, TG, SBP, and FPG. ^#^Variables in the model: sex, age, family history of diabetes, smoking, drinking, physical inactive, BMI, TC, TG, SBP, and categories of pre-DM (i-IFG, i-IGT, and IFG-IGT). FPG or different categories of pre-DM were significant independent risk factors for the conversions between different glycemic statuses (data not shown). NGT: normal glucose tolerance; pre-DM: prediabetes; DM: diabetes mellitus; BMI: body mass index; SBP: systolic blood pressure; TC: total cholesterol; TG: triglyceride; ORs: odds ratios of per one standard deviation (SD) unit increment in these variables.

For individuals with NGT at Baseline, multivariate analysis identified BMI, serum TC, serum TG, SBP, and FPG as significant risk factors of progression to prediabetes, while sex, serum TC, serum TG, SBP, and FPG were associated with progression to diabetes (Table 2). Baseline factors significantly associated with progression from prediabetes to diabetes included serum TG, SBP, and the three categories of prediabetes (Table 2). When comparing the ORs per one SD increment in the modifiable metabolic risk factors (BMI, TC, TG, and SBP), serum TG was found to be the strongest risk factor for the development of diabetes for individuals with either NGT or prediabetes at Baseline. A one SD increase in serum TG was associated with a 1.29- (95% CI 1.10–1.52; *P* = 0.002) or 1.12- (95% CI 1.01–1.27; *P* = 0.039) fold higher risk of diabetes for individuals with NGT or prediabetes at Baseline, respectively (Figure 1). Compared with subjects with normal baseline serum TG levels, elevated serum TG (>2.25 mmol/L) was associated with 1.97 (95% CI 1.23–3.16; *P* = 0.005) and 1.36 (95% CI 1.13–1.87; *P* = 0.024) times higher risk of developing diabetes from NGT or prediabetes, respectively (Table 2).

### 3.5. Diabetes Risk Was Modified according to Changes in Serum TG over Time

**Figure 2:**
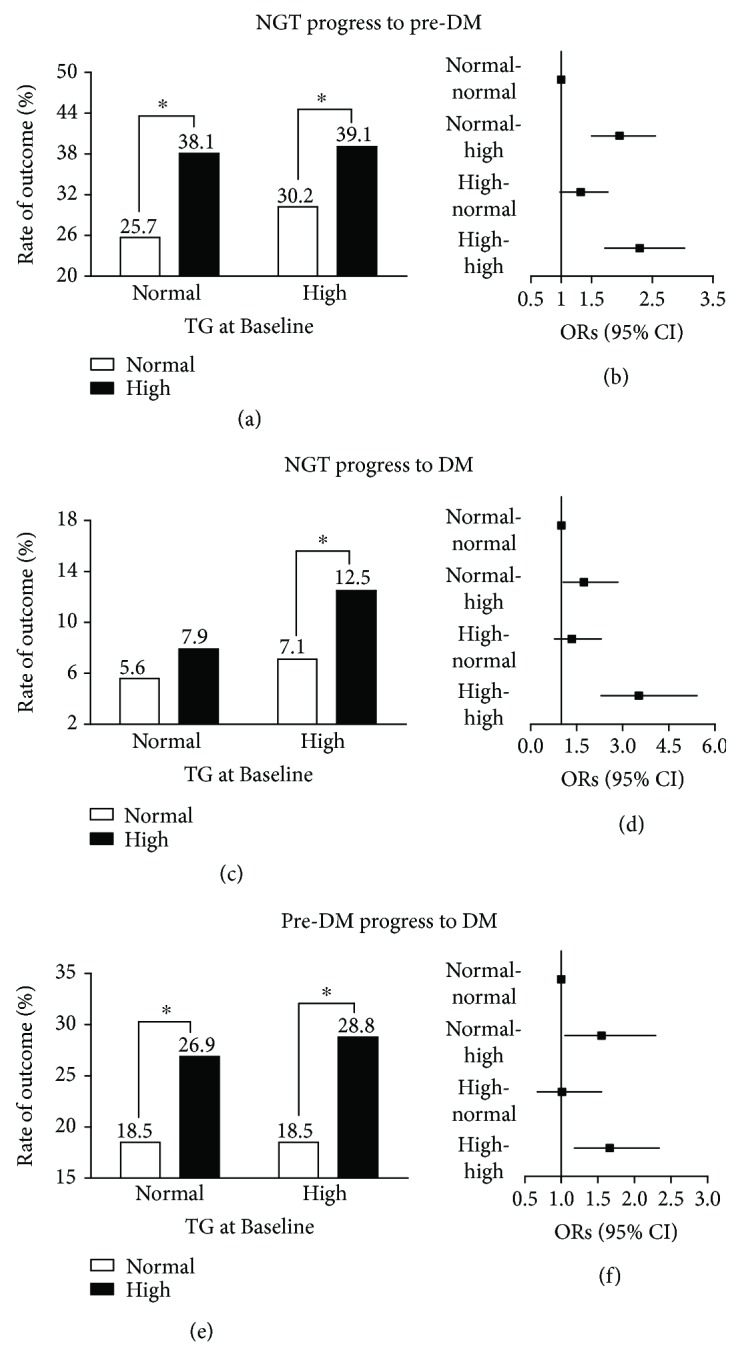
Association between changes in serum triglyceride levels and future incidence of dysglycemia. NGT: normal glucose tolerance; pre-DM: prediabetes; DM: diabetes mellitus; TG: triglyceride. Black bars: high TG at 3-year follow-up; white bars: normal TG at 3-year follow-up. (a, c, e) Percentage of corresponding outcomes of each group as classified according to serum TG levels at Baseline and 3-year follow-up. (b, d, f) Multivariate logistic regression model comparing ORs for corresponding outcomes associated with each group, taking the normal–normal group as the reference (OR = 1), after adjusting for age, sex, family history of diabetes, baseline fasting plasma glucose level (for individuals with NGT at Baseline) or categories of pre-DM (for individuals with pre-DM at Baseline), and changes in body mass index. ^∗^ *P* < 0.05 for comparison between the two groups.

With TG demonstrated to be the strongest risk factor for incident diabetes, we next evaluated the association between changes in serum TG during the 3-year follow-up and glycemic outcome using multivariate analysis. Serum TG levels at Baseline and at 3-year follow-up were divided into two categories, “normal” (TG < 1.7 mmol/L) or “high” (TG ≥ 1.7 mmol/L). Accordingly, patients were grouped as either normal–normal (i.e., TG < 1.7 mmol/L at Baseline and 3-year follow-up), normal–high, high–normal, or high–high. Among subjects with NGT at Baseline, those in the normal–high TG group were 1.96 (95% CI 1.50–2.55; *P* < 0.001) and 1.73 (95% CI 1.06–2.84; *P* = 0.029) times more likely to develop prediabetes or diabetes, respectively, compared with those in the normal–normal TG group (Figures 2(a)–2(d)). Remarkably, in participants with high TG at Baseline, normal TG at 3-year follow-up was associated with decreased risk of prediabetes or diabetes; ORs for prediabetes and diabetes were 2.29 (95% CI 1.72–3.03; *P* < 0.001) and 3.52 (95% CI 2.29–5.41; *P* < 0.001), respectively, in the high–high group compared with the normal–normal group but decreased to 1.32 (95% CI 0.98–1.77; *P* = 0.064) and 1.34 (95% CI 0.78–2.30; *P* = 0.286), respectively, for the high–normal group (Figures 2(a)–2(d)). A similar association between changes in TG and risk of progression to diabetes was observed for subjects with prediabetes at Baseline (Figures 2(e) and 2(f)).

### 3.6. BMI Was Most Prominent for Regression from Prediabetes to NGT

**Figure 3:**
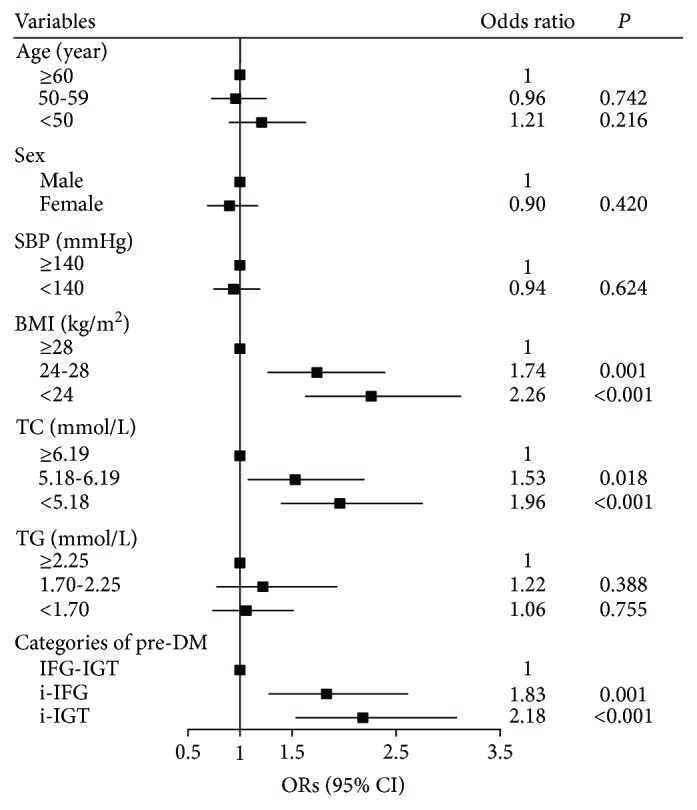
Predictors of regression from prediabetes to normal glucose tolerance at the 3-year follow-up. ORs (95% CI): odds ratios and 95% confidence intervals calculated in the multivariate logistic model. Variables in the model: sex, age, family history of diabetes, smoking, drinking, physical inactive, SBP, BMI, TC, TG, and categories of Pre-DM. Family history of diabetes, smoking, drinking, and physical inactivity were not significantly associated with the regression from pre-DM to NGT and were not shown in the figure. SBP: systolic blood pressure; BMI: body mass index; TC: total cholesterol; TG: triglyceride; Pre-DM: prediabetes; IFG-IGT: combined status of IFG and IGT; i-IFG: isolated impaired fasting glucose; i-IGT: isolated impaired glucose tolerance.

Figure 3 shows the contributions of different variables on the regression from prediabetes to NGT. Lower BMI, lower serum TC, and i-IGT or i-IFG compared with IFG-IGT were the predictors for regression from prediabetes to NGT. BMI was identified as the strongest factor associated with reversion to NGT; a one SD increment in baseline BMI was associated with a 20% decrease (OR 0.80; 95% CI 0.74–0.91; *P* < 0.001) in the rate of regression to NGT (Figure 1). The other remarkable finding was that elevated serum TC was also an independent factor that impedes the regression from prediabetes to NGT; subjects with prediabetes who had normal serum TC at Baseline were 1.96 (95% CI 1.40–2.75; *P* < 0.001) times more likely to revert to NGT compared with those with elevated serum TC level (Figure 3).

## 4. Discussion

In this population-based, longitudinal cohort study, we documented the glycemic outcomes for 4,577 nondiabetic subjects from rural China after a 3-year follow-up, using OGTT to determine the glycemic status. This represents the largest such study conducted in China. This study also systemically investigated risk factors for the conversions among NGT, prediabetes, and diabetes and found that the spectrum of risk factors differed at different stages of pathophysiology of type 2 diabetes. Specifically, serum TG was identified as the strongest independent risk factor for diabetes versus other modifiable metabolic risk factors. Meanwhile, as serum TG changed over time, the risk of diabetes was correspondingly modified. On the other hand, BMI was important for the remission from prediabetes to NGT, but not so crucial for progression after the other states.

The incidence rate of dysglycemia in the Chinese population has been infrequently reported [21]. We observed that over a median follow-up of 3.1 years, 28.7% of individuals aged ≥40 years with NGT at Baseline progressed to prediabetes, representing an incidence rate of 93.6 per 1,000 person-years. This figure is comparatively high compared with reports from other populations, such as the Danish population from the Inter99 study (21.0 per 1,000 person-years) [22] and Asian Indians from the CURES study (51.7 per 1,000 person-years) [12]. The older age of the present study population may have contributed to the higher incidence of prediabetes, but it likely also reflects the rapid increase in prevalence of prediabetes in China and highlights the necessity of implementing preventive strategies prior to the onset of prediabetes to control the epidemic of diabetes.

It has been established that the prediabetic states of i-IFG, i-IGT, and IFG-IGT represent distinct pathophysiological changes, including different degrees of insulin sensitivity and insulin secretion, as well as secretion of gut incretin hormones [23, 24]. However, there is still controversy about which of these groups of prediabetes should be prioritized for preventive intervention [25, 26]. In our study, we classified prediabetes into i-IFG, i-IGT, and IFG-IGT, which enabled differentiation of glycemic outcomes of these three categories. Interestingly, we observed that subjects with i-IFG were at higher risk of diabetes and less likely to revert to NGT compared with subjects with i-IGT. This result was in accordance with previous studies conducted in European populations [11, 27]. The findings indicate that a more intensive monitoring of glycemic status should be recommended for patients with i-IFG rather than those with i-IGT.

In this study, FPG or categories of prediabetes, serum TG, and SBP were recognized as risk factors of incident diabetes for individuals with either NGT or prediabetes at Baseline. Notably, comparison of the ORs of a per one SD change in serum TG and SBP revealed that serum TG was the strongest modifiable metabolic risk factor for diabetes. In accordance with our results, prior studies [28, 29] reported that elevated serum TG was associated with a higher risk of diabetes. The available experimental and clinical evidence suggested that TGs per se may directly contribute to disorders of glucose metabolism [30]. Given that the prevalence of hypertriglyceridemia has been estimated to be as high as 21.6–33.5%, varying by different populations [31, 32], it is plausible that the high prevalence of hypertriglyceridemia potentially contributes to the high incidence of diabetes worldwide. Fortunately, our study demonstrated that the rate of progression to diabetes is lower in people who are able to lower elevated TG levels to a normal level, which is in accordance with a previous study conducted in healthy males [33]. Moreover, a randomized controlled trial [34] has proven that controlling serum TG by fenofibrate could ameliorate the natural course of prediabetes, and the effect was similar to metformin and superior to diet control. Based on these results, hypertriglyceridemia should be regarded as a public health problem, and efforts to monitor and control serum TG may introduce substantial benefits for both individuals and the whole society.

It is known that spontaneous regression from prediabetes to NGT can occur, and in the present analysis, 33.1% of individuals with prediabetes at Baseline reverted to normoglycemia after a 3-year follow-up. We demonstrated that factors negatively associated with reversion from prediabetes to NGT included elevated BMI and elevated serum TC. In addition, serum TC, serum TG, SBP, and BMI were independently associated with the progression from NGT to prediabetes. Overall, these findings show that serum TC plays an important role in the bidirectional conversion between NGT and prediabetes. To our knowledge, it is the first time that serum cholesterols have been reported to predict the reversion process from prediabetes to NGT independently. In agreement with our results, Janghorbani and Amini [35] observed that serum cholesterol was significantly decreased from baseline in people with prediabetes who regressed to NGT over a 2-year period, supporting the association between cholesterols and the regression to NGT. Taken together, serum cholesterol may be a useful tool for making preventive recommendations for patients with prediabetes.

Generally, obesity, either with elevated BMI or waist circumference, was regarded as the primary risk factor of diabetes [36]. In accordance with previous studies, we also found that BMI was independently associated with the mutual conversion between NGT and prediabetes. However, BMI failed to be an independent predictor of incident diabetes in the multivariate model. The different roles of BMI in different paths of glycemic status conversions might be owing to the distinct pathophysiology in the multistage of diabetes development [37]. In the early stage of diabetes development, insulin resistance is the dominating pathological mechanism, which is more related to obesity [38]. In the late stage of progression to diabetes, the disturbance of glycemic homeostasis is mainly due to the decreased *β*-cell function, in which hyperlipidemia is more important [39].

The strengths of our study include the prospective cohort design and relatively large sample size. We systemically evaluated the risk factors for the conversions among the three stages of diabetes, with each path of conversion studied in the corresponding subcohort (Figure 1). What is more, the findings of our study have important public health implications. First of all, we illuminated the important role of serum lipids in the disease course of diabetes, which suggested that adequate control of hyperlipidemias should be regarded as an indispensable strategy in preventing diabetes. Since the spectrum of risk factors for the divergent conversions among the three stages differed, clinicians or public health workers should provide personalized preventive strategies for individuals with different basal glycemic statuses. On the other hand, for subjects with multiple risk factors for diabetes, the risk factor with the strongest association should be taken as the primary target variable to bring under control. This principle is of particular benefit to patients with financial limitations or limited access to medical care.

There are also several limitations to this study which deserve mention. Firstly, we used follow-up data from one time point at 3 years, which precludes us from following the dynamic changes of glycemic status over the long term. Furthermore, there was a lack of information about changes in lifestyle or interventions for prediabetes initiated during the follow-up, which might confound the predictive effect of baseline characteristics. In addition, for ethical restrictions, we were not able to evaluate the natural glycemic outcome and associated factors of individuals with diabetes at Baseline. Family history of diabetes, as one of the known independent risk factor for diabetes, was not associated with the conversions of glycemic status in this study. This variable of being self-reported retrospective data might account for the absence of positive association.

In summary, this study conducted in a rural Chinese population found a high incidence of prediabetes over a 3-year follow-up in subjects with NGT at Baseline, supporting the implementation of preventive strategies prior to the onset of prediabetes to control the epidemic of diabetes. We observed that the spectrum of risk factors varied at different stages of pathophysiology of type 2 diabetes, suggesting personalized preventive strategies for individuals with different basal glycemic statuses. On the other hand, hyperlipidemias were important and modifiable risk factors associated with type 2 diabetes. Adequately controlling hyperlipidemias is indispensable in disposing of the burden of diabetes.

## Figures and Tables

**Table 1:** Incidence rates of prediabetes and diabetes.

Glycemic status at Baseline	*n* ^∗^	Person-years	Glycemic status at 3-year follow-up	Outcomes (*n*)	Incidence rate (per 1,000 person-years)	
					Crude rate (95% CI)	Age- and sex-standardized rates
NGT	2,833	8,499	i-IGT	609	71.6 (66.2–77.1)	68.3
		8,499	i-IFG	96	11.3 (9.5–13.5)	12.9
		8,499	IFG-IGT	108	12.7 (10.3–15.1)	12.5
		8,499	Pre-DM	813	95.7 (89.4–101.9)	93.6
		8,499	DM	188	22.1 (19.0–25.2)	24.2
		8,499	Pre-DM or DM	1,001	117.8 (111.0–124.6)	117.8

i-IFG	640	1,920	IFG-IGT	75	39.1 (30.4–47.7)	37.3
		1,920	DM	144	75.0 (63.2–86.8)	70.5
		1,920	IFG-IGT or DM	219	114.4 (99.8–128.3)	107.8

i-IGT	790	2,370	IFG-IGT	54	22.8 (16.8–28.8)	21.7
		2,370	DM	129	54.4 (45.3–63.6)	56.9
		2,370	IFG-IGT or DM	183	77.2 (66.5–88.0)	78.6

IFG-IGT	314	942	DM	95	100.8 (81.6–120.0)	104.3

Pre-DM	1,744	5,232	DM	368	70.3 (63.4–77.3)	70.3

NGT, Pre-DM	4,577	13,731	DM	556	40.5 (37.2–43.8)	42.2

^∗^Number of individuals at Baseline. NGT: normal glucose tolerance; i-IFG: isolated impaired fasting glucose; i-IGT: isolated impaired glucose tolerance; IFG-IGT: combined status of IFG and IGT; Pre-DM: prediabetes; DM: diabetes mellitus.

**Table 2:** Baseline factors associated with progression to prediabetes and diabetes.

Variables	NGT to pre-DM		NGT to DM		Pre-DM to DM	
	OR (95% CI)	*P* value	OR (95% CI)	*P* value	OR (95% CI)	*P* value
Female	1.10 (0.90–1.36)	0.353	0.65 (0.45–0.93)	0.017	0.97 (0.73–1.30)	0.847
Age (years)		0.232		0.368		0.363
<50	1		1		1	
50–60	1.19 (0.97–1.46)	0.090	1.22 (0.83–1.80)	0.304	1.03 (0.73–1.45)	0.872
≥60	1.09 (0.86–1.38)	0.485	1.36 (0.89–2.08)	0.151	1.24 (0.87–1.77)	0.235
BMI (kg/m^2^)		0.011		0.195		0.890
<24	1		1		1	
24–28	1.18 (0.97–1.44)	0.093	1.20 (0.84–1.72)	0.317	1.04 (0.77–1.41)	0.793
≥28	1.45 (1.14–1.85)	0.003	1.51 (0·97–2.35)	0.072	1.09 (0.77–1.53)	0.629
TC (mmol/L)		<0.001		0.045		0.601
<5.18	1		1		1	
5.18–6.19	1.50 (1.24–1.83)	<0.001	1.25 (0.87–1.80)	0.221	1.15 (0.85–1.54)	0.369
≥6.19	1.50 (1.15–1.97)	0.003	1.66 (1.05–2.61)	0.029	0.99 (0.70–1.39)	0.943
TG (mmol/L)		0.071		0.015		0.046
<1.70	1		1		1	
1.70–2.25	1.14 (0.85–1.53)	0.388	1.38 (0.84–2.26)	0.203	0.94 (0.62–1.40)	0.745
≥2.25	1.40 (1.04–1.87)	0.025	1.97 (1.23–3.16)	0.005	1.36 (1.13–1.87)	0.024
SBP ≥ 140 mmHg	1.26 (1.05–1.51)	0.013	1.91 (1.39–2.64)	<0.001	1.33 (1.03–1.75)	0.029
Positive FHD	1.16 (0.59–2.30)	0.666	0.78 (0.18–3.41)	0.741	0.43 (0.14–1.30)	0.136
Drinking	1.04 (0.82–1.31)	0.776	0.99 (0.65–1.50)	0.956	0.79 (0.55–1.11)	0.175
Smoking	1.04 (0.78–1.39)	0.807	1.18 (0.73–1.90)	0.503	1.43 (0.95–2.15)	0.089
Physical inactive	1.04 (0.88–1.24)	0.639	1.16 (0.84–1.59)	0.366	1.12 (0.86–1.45)	0.410
FPG (mmol/L)^∗^	1.62 (1.28–2.06)	<0.001	1.78 (1.13–2.76)	0.012	—	—
Categories of pre-DM						0.009
i-IGT	—	—	—	—	1	
i-IFG	—	—	—	—	1.41 (1.05–1.90)	0.022
IFG-IGT	—	—	—	—	1.63 (1.17–2.29)	0.004

^∗^Included in the model as a continuous variable. OR (95% CI): odds ratios and 95% confidence intervals calculated in the multivariate logistic model. Variables in the model: sex, age, family history of diabetes, smoking, drinking, physical inactive, SBP, BMI, TC, TG, FPG (for individuals with NGT at Baseline), or categories of pre-DM (for individuals with pre-DM at Baseline). NGT: normal glucose tolerance; Pre-DM: prediabetes; DM: diabetes mellitus; BMI: body mass index; TC: total cholesterol; TG: triglyceride; SBP: systolic blood pressure; FHD: family history of diabetes; FPG: fasting plasma glucose; i-IGT: isolated impaired glucose tolerance; i-IFG: isolated impaired fasting glucose; IFG-IGT: combined status of IFG and IGT.

## Data Availability

The data used to support the findings of this study are available from the corresponding author upon request.

## Supplementary Materials

Supplementary Materials The supplementary material file includes the following items: (1) details about the authors, (2) details about the study measurements, (3) Supplementary Figure 1: participant selection and follow-up flow diagram, (4) Supplementary Table 1: baseline characteristics of individuals with and without follow-up, (5) Supplementary Table 2: glycemic status at Baseline and 3-year follow-up, (6) Supplementary Table 3: baseline characteristics of the participants based on their glycemic status at Baseline and 3-year follow-up.
