# Supplementary material for: Different Contributions of Dyslipidemia and Obesity to the Natural History of Type 2 Diabetes: 3-Year Cohort Study in China
Source: J Diabetes Res. 2019 Mar 4;2019:4328975. doi: 10.1155/2019/4328975 (PMC6425409; doi:10.1155/2019/4328975)
Supplement: Supplementary Materials — The supplementary material file includes the following items: (1) details about the authors, (2) details about the study measurements, (3) Supplementary Figure 1: participant selection and follow-up flow diagram, (4) Supplementary Table 1: baseline characteristics of individuals with and without follow-up, (5) Supplementary Table 2: glycemic status at Baseline and 3-year follow-up, (6) Supplementary Table 3: baseline characteristics of the participants based on their glycemic status at Baseline and 3-year follow-up. [file 4328975.f1.pdf]

REACTION Study Group

Steering Committee: Guang Ning (Principle Investigator), National Clinical Research Center for Metabolic Diseases, Rui Jin Hospital, Shanghai Jiao Tong University School of Medicine, Shanghai, China; Yiming Mu, People's Liberation Army General Hospital, Beijing, China; Jiajun Zhao, Shandong Provincial Hospital, Jinan, China; Weiqing Wang, National Clinical Research Center for Metabolic Diseases, Rui Jin Hospital, Shanghai Jiao Tong University School of Medicine, Shanghai, China; Chao Liu, Jiangsu Province Hospital on Integration of Chinese and Western Medicine, Nanjing, China; Yufang Bi, National Clinical Research Center for Metabolic Diseases, Rui Jin Hospital, Shanghai Jiao Tong University School of Medicine, Shanghai, China; Donghui Li, Department of Gastrointestinal Medical Oncology, the University of Texas MD Anderson Cancer Center, Houston, Texas, USA; Shenghan Lai, Johns Hopkins University School of Medicine, Baltimore, Maryland, USA; Zachary T. Bloomgarden, Mount Sinai School of Medicine, New York, USA.

Working Group: Weiqing Wang, Yufang Bi, Jieli Lu, National Clinical Research Center for Metabolic Diseases, Rui Jin Hospital, Shanghai Jiao Tong University School of Medicine, Shanghai, China; Yiming Mu, People's Liberation Army General Hospital, Beijing, China; Jiajun Zhao, Shandong Provincial Hospital, Jinan, China; Chao Liu, Jiangsu Province Hospital on Integration of Chinese and Western Medicine, Nanjing, China; Lulu Chen, Wuhan Xiehe Hospital, Huazhong University of Science and Technology School of Medicine, Wuhan, China; Lixin Shi, Affiliated Hospital of Guiyang Medical College, Guiyang, China; Qiang Li, The Second Affiliated Hospital of Haerbin Medical University, Haerbin, China; Tao Yang, The First Affiliated Hospital with Nanjing Medical University, Jiangsu Province Hospital, Nanjing, China; Li Yan, Sun Yat-sen Memorial Hospital, Sun Yat-sen University, Guangzhou, China; Qin Wan, The Affiliated Hospital of Luzhou Medical College, Luzhou, China; Shengli Wu, Xinjiang Kelamayi Peoples Hospital, Kelamayi, China; Guixia Wang,

The First Hospital of Jilin University, Changchun, China; Zuojie Luo, The First Affiliated Hospital of Guangxi Medical University, Nanning, China; Xulei Tang, The First Hospital of Lanzhou University, Lanzhou, China; Gang Chen, Fujian Provincial Hospital, Fujian Medical University, Fuzhou, China; Yanan Huo, Jiangxi People's Hospital, Nanchang, China; Zhengnan Gao, Dalian Municipal Central Hospital, Dalian, China; Qing Su, Xinhua Hospital, Shanghai Jiao Tong University School of Medicine, Shanghai, China; Zhen Ye, Zhejiang Center for Disease Control, China; Youmin Wang, The First Affiliated Hospital of Anhui Medical University, Hefei, China; Guijun Qin, The First Affiliated Hospital of Zhengzhou University, Zhengzhou, China; Huacong Deng, The First Affiliated Hospital of Chongqing Medical University, Chongqing, China; Xuefeng Yu, Wuhan Tongji Hospital, Huazhong University of Science and Technology School of Medicine, Wuhan, China; Feixia Shen, The First Affiliated Hospital of Wenzhou Medical University, The First Provincial Wenzhou Hospital of Zhejiang, Wenzhou, China; Li Chen, Qilu Hospital, University of Shandong School of Medicine, Jinan, China

Study measurements

All study measurements were collected both at Baseline and 3-year follow-up visit. Trained staff collected information about social-demographic characteristics, medical history and family history using a standard questionnaire and face-to-face interviews. Current smoking was defined as currently smoking cigarettes at the time of the survey and having smoked >100 cigarettes in their lifetime. Current drinking was defined as alcohol intake more than once per month during the past 12 months. Physical activity was evaluated by the Global Physical Activity Questionnaire and was quantified by the metabolic equivalent (MET). Physical inactive was defined as the total MET did not reach the World Health Organization (WHO) recommendation (600 minutes per week).

Clinical assessments included measurements of weight, height, waist circumference and blood pressure. Weight (kilograms) and standing height (meters) were measured in light clothing and without shoes. Waist circumference was measured to the nearest 0.1 cm at umbilical level in a standing position. Blood pressure was measured three times consecutively at the non-dominant arm with a 3 minute interval after a 5 minute rest using an electronic sphygmomanometer (OMRON Model HEM-725FUZZY, Omron Company, Dalian, China) with the participant in a sitting position. The average value of the three readings was used for data analysis.

Venous blood samples were drawn between 8:00 am and 10:00 am after an overnight fast for at least 10 hours. Participants without a self-reported history of diabetes underwent a standard OGTT (75 g anhydrous glucose in 250 ml water), and plasma glucose was measured at 0 hours (FPG) and 2 hours (2hPG) during the OGTT using the glucose oxidase method, within 2 hours of blood sample collection. Fasting serum lipid profiles were quantified using an auto-analyzer (ARCHITECT c16000 System, Abbott Laboratories, IL, USA). Capillary

haemoglobin A_{1c} (HbA_{1c}) was measured using high performance liquid chromatography (VARIANT™ II and D-10™ Systems, BIO-RAD, Hercules, CA, USA) with capillary blood samples collected using the Hemoglobin Capillary Collection System (Bio-Rad Laboratories, Hercules, CA, USA).

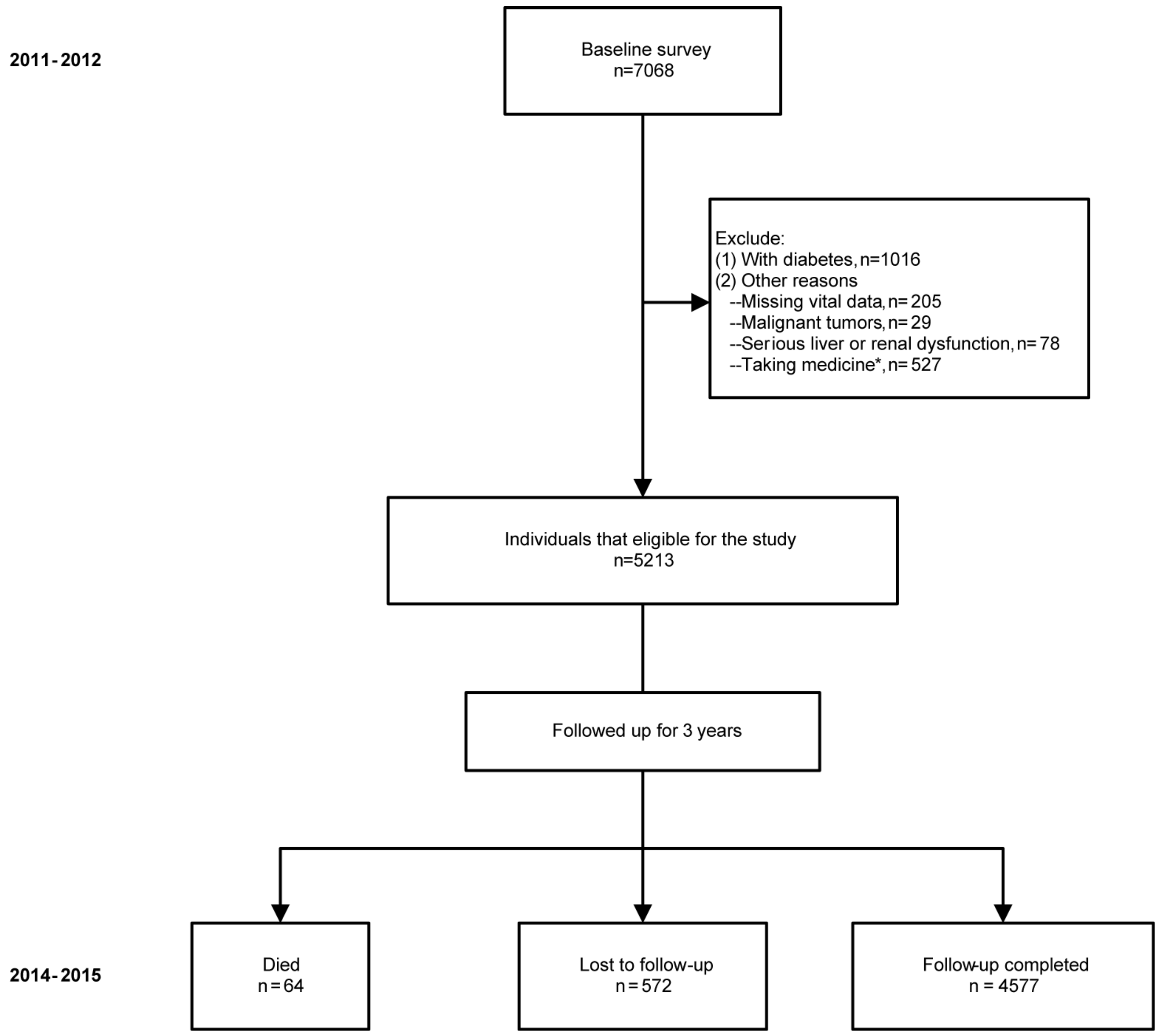

Supplementary Figure 1—Participant selection and follow-up flow diagram

* Details are given in the Study Population section.

Supplementary Table 1─Baseline characteristics of individuals with and without follow-up

**Variables**	**Followed up** ***n*** **= 4,577**	**Lost to follow up or dead** ***n*** **= 636**	***p*** **value**
Male, n (%)	1,762 (38.5)	255 (40.1)	0.438
Age (years)	54.63 ± 8.45	54.64 ± 9.33	0.325
Weight (kg)	64.43 ± 10.74	63.96 ± 11.25	0.052
BMI (kg/m^2)	25.17 ± 3.69	24.84 ± 3.74	0.391
Waist (cm)	87.02 ± 10.33	86.62 ± 10.29	0.203
TC (mmol/L)	5.07 ± 1.13	5.03 ± 0.99	0.215
TG (mmol/L)	1.11 (0.78)	1.09 (0.76)	0.353
HDL-C (mmol/L)	1.45 ± 0.36	1.44 ± 0.36	0.546
LDL-C (mmol/L)	2.99 ± 0.89	2.96 ± 0.80	0.147
SBP (mmHg)	137.74 ± 20.73	136.28 ± 21.08	0.961
DBP (mmHg)	81.09 ± 11.63	80.65 ± 11.77	0.980
FPG (mmol/L)	5.66 ± 0.53	5.64 ± 0.52	0.372
2hPG (mmol/L)	7.21 ± 1.74	7.15 ± 1.79	0.359
HbA_{1c} (%)	5.79 ± 0.40	5.75 ± 0.40	0.189
HbA_{1c} (mmol/mol)	40	39	─
Positive FHD, n(%)	87 (1.9)	10 (1.6)	0.566
Current smoker, n (%)	705 (15.4)	108 (17.0)	0.304
Current drinker, n (%)	1,213 (26.5)	165 (25.9)	0.765
Physical inactive, n (%)	2069 (45.2)	272 (42.8)	0.247

Values for quantitative data are presented as mean ± standard deviation, or median (inter-quartile range); values for categorical variables are presented as number (percentage).

BMI, body mass index; TC, total cholesterol; TG, triglyceride; HDL-C, high-density lipoprotein cholesterol; LDL-C, low-density lipoprotein cholesterol; SBP, systolic blood pressure; DBP, diastolic blood pressure; FPG, fasting plasma glucose; 2hPG, 2h plasma glucose; FHD, family history of diabetes.

Supplementary Table 2─Glycemic status at Baseline and 3-year follow-up

**Glycemic status at Baseline**	***n*** [*]	**Glycemic status at 3-year follow-up**	**Outcomes**	
			n	**Rate (%)**
NGT	2,833	NGT	1,832	64.7
		i-IGT	609	21.5
		i-IFG	96	3.4
		IFG-IGT	108	3.8
		Pre-DM	813	28.7
		DM	188	6.6
		Pre-DM, or DM	1,001	35.3
Pre-DM	1,744	NGT	577	33.1
		Pre-DM	799	45.8
		DM	368	21.1
I-IFG	640	NGT	205	32.0
		i-IGT	111	17.3
		i-IFG	105	16.5
		IFG-IGT	75	11.7
		DM	144	22.5
		IFG-IGT, or DM	219	34.2
I-IGT	790	NGT	311	39.4
		i-IGT	260	32.9
		i-IFG	36	4.6
		IFG-IGT	54	6.8
		DM	129	16.3
		IFG-IGT, or DM	183	23.2
IFG-IGT	314	NGT	61	19.4
		i-IGT	67	21.3
		i-IFG	35	11.2
		IFG-IGT	56	17.8
		DM	95	30.3

[*] Number of individuals at Baseline.

NGT, normal glucose tolerance; i-IGT, isolated impaired glucose tolerance; i-IFG, isolated impaired fasting glucose; IFG-IGT, combined status of IFG and IGT; Pre-DM, pre-diabetes; DM, diabetes mellitus.

Supplementary Table 3. Baseline characteristics of the participants based on their glycemic status at Baseline and 3-year follow-up

Variables	NGT at Baseline (*n* = 2,833)				Pre-DM at Baseline (*n* = 1,744)			
	Remained as NGT	Progressed to pre-DM	Progressed to DM	*P* value	Regressed to NGT	Remained as pre-DM	Progressed to DM	*P* value
n (%)	1,832 (64.7)	813 (28.7)	188 (6.6)		577 (33.1)	799 (45.8)	368 (21.1)	
Male, n (%)	650 (35.5)	286 (35.2)	91 (48.4)*	0.002	256 (44.4)	323 (40.4)	158 (42.9)	0.329
Age (years)	53.42 ± 8.39	54.35 ± 8.10*	54.85 ± 8.29	0.005	55.58 ± 8.86	55.74 ± 8.09	57.30 ± 8.54†‡	0.004
BMI (kg/m^2)	24.69 ± 3.45	25.37 ± 3.66*	25.42 ± 3.61	<0.001	24.79 ± 3.40	25.67 ± 3.55†	25·92 ± 3.76†	<0.001
Waist (cm)	86.07 ± 10.05	87.05 ± 10.39*	88.63 ± 12.30*	0.001	86.93 ± 10.26	87.98 ± 10.38	88.98 ± 10.12†	0.011
TC (mmol/L)	4.88 ± 1.07	5.16 ± 1.11*	5.16 ± 1.17*	<0.001	4.99 ± 1.12	5.30 ± 1.19†	5.37 ± 1.13†	<0.001
TG (mmol/L)	1.02 (0.67)	1.15 (0.78)*	1.20 (1.06)*	<0.001	1.12 (0.77)	1.25 (0.91)†	1.28 (1.07)†	0.001
HDL-C (mmol/L)	1.45 ± 0.35	1.44 ± 0.34	1.44 ± 0.38	0.903	1.44 ± 0.38	1.47 ± 0.38	1.43 ± 0.35	0.123
LDL-C (mmol/L)	2.85 ± 0.84	3.07 ± 0.89*	3.03 ± 0.95*	<0.001	2.95 ± 0.84	3.15 ± 0.95†	3.24 ± 0.93†	<0.001
SBP (mmHg)	133.42 ± 19.80	138.11 ± 20.69*	141.23 ± 21.12*	<0.001	139.01 ± 20.12	141.60 ± 21.07	146.13 ± 20.49†‡	<0.001
DBP (mmHg)	79.44 ± 11.29	80.10 ± 11.34	82.40 ± 12.79*	0.002	83.29 ± 11.42	82.71 ± 11.85	83.82 ± 11.29	0.300
FPG (mmol/L)	5.40 ± 0.37	5.49 ± 0.38*	5.52 ± 0.43*	<0.001	5.88 ± 0.53	6.03 ± 0.52†	6.18 ± 0.52†‡	<0.001
2hPG (mmol/L)	6.07 ± 1.07	6.21 ± 1.11	6.25 ± 1.05*	0.028	8.39 ± 1.42	8.53 ± 1.41†	8.85 ± 1.49†‡	<0.001
HbA_{1c} (%)	5.68 ± 0.35	5.77 ± 0.34*	5.86 ± 0.49*#	<0.001	5.81 ± 0.35	5.90 ± 0.44†	6.12 ± 0.44†‡	<0.001
HbA_{1c} (mmol/mol)	39	40	41	–	40	41	43	–
Positive FHD, n (%)	27 (1.5)	13 (1.6)	2 (1.1)	0.860	21 (3.6)	22 (2.8)	4 (1.1)	0.061
Current Smoker, n (%)	267 (14.6)	116 (14.3)	38 (20.2)	0.100	95 (16.5)	123 (15.4)	65 (17.7)	0.610
Current Drinker, n (%)	447 (24.4)	199 (24.5)	54 (28.7)	0.418	183 (31.7)	228 (28.5)	101 (27.4)	0.293
Physical inactive, n (%)	779 (42.5)	361 (44.4)	83 (44.1)	0.640	291 (50.4)	374 (46.8)	181 (49.2)	0.397

[*] $P < 0.05$ compared with individuals who remained as NGT; [#] $P < 0.05$ compared with those who progressed to pre-DM; [†] $P < 0.05$ compared with individuals who regressed to NGT; [‡] $P < 0.05$ compared with those who remained as pre-DM.

Values for quantitative data are presented as mean ± standard deviation, or median (inter-quartile range); values for categorical variables are presented as number (percentage).

NGT, normal glucose tolerance; Pre-DM, pre-diabetes; DM, diabetes mellitus; BMI, body mass index; TC, total cholesterol; TG, triglyceride; HDL-C, high-density lipoprotein cholesterol; LDL-C, low-density lipoprotein cholesterol; SBP, systolic blood pressure; DBP, diastolic blood pressure; FPG, fasting plasma glucose; 2hPG, 2h plasma glucose; FHD, family history of diabete
